# Expression of Heart Development Protein With EGF‐Like Domains 1 (HEG1) Decorated With Low‐Sulfated Keratan Sulfate in Human Malignant Pleural Mesothelioma

**DOI:** 10.1111/pin.70033

**Published:** 2025-06-17

**Authors:** Koki Nakashima, Hitomi Hoshino, Zui Zhang, Tomoya O. Akama, Nobuyuki Kondo, Seiki Hasegawa, Yoshitaka Sekido, Mana Fukushima, Tamotsu Ishizuka, Motohiro Kobayashi

**Affiliations:** ^1^ Department of Pathology, Faculty of Medical Sciences University of Fukui Eiheiji Japan; ^2^ Department of Respiratory Medicine, Faculty of Medical Sciences University of Fukui Eiheiji Japan; ^3^ Department of Biochemistry Nagoya University Graduate School of Medicine Nagoya Japan; ^4^ Department of Pharmacology Kansai Medical University Hirakata Japan; ^5^ Division of Thoracic Surgery, Department of Surgery Hyogo College of Medicine Nishinomiya Japan; ^6^ Division of Cancer Biology, Aichi Cancer Center Research Institute Nagoya Japan

**Keywords:** heart development protein with EGF‐like domains 1 (HEG1), low‐sulfated keratan sulfate, malignant pleural mesothelioma, protein glycosylation

## Abstract

The glycoform of heart development protein with EGF‐like domains 1 (HEG1) recognized by the SKM9‐2 monoclonal antibody is a useful diagnostic marker for malignant pleural mesothelioma (MPM). The putative glycoform includes core 2 *O*‐glycans carrying sialyl poly‐*N*‐acetyllactosamine (LacNAc), but sulfation modifications are undetermined. Since sialyl 6‐sulfo LacNAc‐capped core 2 *O*‐glycans are expressed in MPM and their structure overlaps with low‐sulfated keratan sulfate (KS), we asked whether low‐sulfated KS is expressed in MPM and whether HEG1 is decorated with low‐sulfated KS. We performed immunohistochemical analysis of 41 MPM cases using anti‐KS monoclonal antibodies and endoglycosidases, reversed‐phase ion‐pair high‐performance liquid chromatography analysis of KS/sulfated LacNAc isolated from human pleural tissue, and western blot analysis of HEG1·IgG recombinant fusion proteins secreted from low‐sulfated KS‐expressing human embryonic kidney cells. Most MPM tissues were stained with anti‐low‐sulfated KS antibodies and staining was eliminated by endo‐β‐galactosidase and keratanase II but not by peptide‐*N*‐glycosidase F. Disaccharide composition analysis revealed that mono‐sulfated LacNAc disaccharide and di‐sulfated LacNAc disaccharide accounted for 83.1% and 16.9% of pleural KS/sulfated LacNAc, respectively. Western blot analysis of HEG1·IgG glycoforms indicated that HEG1 functions as a core protein for low‐sulfated KS. Thus, HEG1 protein decorated with low‐sulfated KS is expressed in MPM.

## Graphical abstract

The purpose of this study is to investigate sulfation modification of the glycan structure decorating HEG1, one of the diagnostic markers of malignant pleural mesothelioma (MPM), in MPM. Immunohistochemical analysis reveals low‐sulfated keratan sulfate (KS) expression, recognized by 294‐1B1 and R‐10G, in MPM tissues with HEG1 expression. Western blot analysis demonstrates that HEG1 functions as a core protein for low‐sulfated KS, suggesting the glycoform may be a potential target for new therapy for MPM.

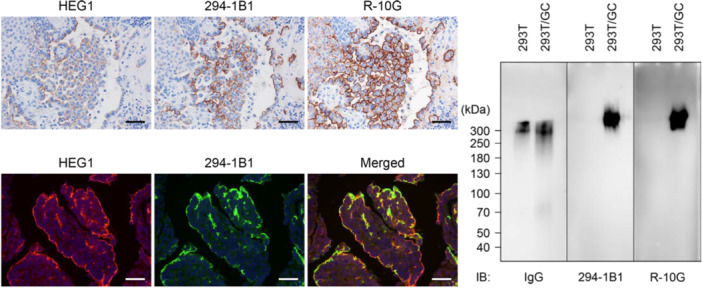

**Abbreviations**
GlcNAc: *N*‐acetylglucosamine
HEG1: heart development protein with EGF‐like domains 1
HEK: human embryonic kidney
HPLC: high‐performance liquid chromatography
HRP: horseradish peroxidase
KS: keratan sulfate
LacNAc: *N*‐acetyllactosamine
MPM: malignant pleural mesothelioma
NIR‐PIT: near‐infrared photoimmunotherapy
PNGase F: peptide‐*N*‐glycosidase F

## 1 Introduction

Malignant pleural mesothelioma (MPM) is a highly aggressive malignant neoplasm of pleural mesothelium caused primarily by asbestos exposure [1, 2]. In recent years, immune checkpoint inhibitors have been developed to treat this malignancy in combination with systemic chemotherapy, but clinical outcomes are insufficient to achieve long‐term survival [3]. Surgical treatment of MPM is also challenging. Pleurectomy/decortication is less invasive to patients than extrapleural pneumonectomy but is less promising in terms of long‐term survival [4]. Thus, there is a need for less invasive therapeutic modalities that can achieve long‐term survival.

Near‐infrared photoimmunotherapy (NIR‐PIT) is a minimally invasive treatment that selectively kills tumor cells by injecting a photoabsorber‐conjugated antibody against a cell surface molecule selectively expressed on cancer cells, followed by near‐infrared irradiation [5]. Nishinaga et al. recently reported that NIR‐PIT with the anti‐podoplanin antibody NZ‐1 showed significant antitumor effects in a mouse MPM model [6]. However, while podoplanin is indeed an established MPM marker, it is also expressed on normal lymphatic endothelial cells and type 1 pneumocytes [6]. Thus, more specific MPM markers would be useful in development of NIR‐PIT for this malignancy.

Tsuji et al. recently developed the monoclonal antibody SKM9‐2, which recognizes the heart development protein with EGF‐like domains 1 (HEG1) protein decorated with sialic acid‐containing glycans [7]. Since SKM9‐2 selectively recognizes MPM tumor cells, particularly those of the epithelioid type, but not normal mesothelial cells or cells in other organs [7], the HEG1 glycoform recognized by SKM9‐2 is currently used as a reliable diagnostic marker for MPM [8] and could also be molecularly targeted as therapy, including in NIR‐PIT [7]. Based on lectin array analysis, Tsuji et al. deduced that the glycan structure decorating HEG1 protein would include sialyl poly‐*N*‐acetyllactosamine (LacNAc) attached to core 2 *O*‐glycans, but did not analyze sulfation modification of this structure.

We and others previously demonstrated that core 2 *O*‐glycans capped with sialyl *N*‐acetylglucosamine (GlcNAc)‐6‐*O*‐sulfated LacNAc are expressed on human MPM tumor cells [9] and mouse normal pleural mesothelial cells [10], using S1 [11] and CL40 monoclonal antibodies [12], respectively. However, the intervening structure between sialyl GlcNAc‐6‐*O*‐sulfated LacNAc at the nonreducing terminal and *N*‐acetylgalactosamine residue attached to core proteins via *O*‐glycosidic bond has not been defined. Since the structure of sialyl GlcNAc‐6‐*O*‐sulfated LacNAc overlaps with that of low‐sulfated keratan sulfate (KS), which consists of repeating GlcNAc‐6‐*O*‐sulfated LacNAc units [13], we hypothesized that at least some S1‐reactive sulfated glycans observed in our previous study are low‐sulfated KS.

Kawabe et al. previously developed the anti‐low‐sulfated KS monoclonal antibody R‐10G, which selectively binds to human embryonic stem and induced pluripotent stem cells [14, 15]. Employing this antibody, Terada‐Uchimura et al. reported that normal mouse mesothelial cells express KS/sulfated LacNAc [16]. Recently, we generated a new anti‐low‐sulfated KS monoclonal antibody, 294‐1B1 [17], and demonstrated that carcinoma cells of human non‐mucinous ovarian [17, 18] and testicular embryonal carcinomas also express low‐sulfated KS [19, 20].

In the present study, employing the above anti‐KS monoclonal antibodies and a series of endoglycosidases, we performed immunohistochemical analysis of 41 MPM cases. We also conducted reversed‐phase ion‐pair high‐performance liquid chromatography (HPLC) analysis of KS/sulfated LacNAc isolated from human pleural tissue. These analyses revealed that core 2 *O*‐glycans carrying low‐sulfated KS are expressed in MPM. Moreover, western blot analysis of an HEG1·IgG recombinant fusion protein indicated that HEG1 serves as a core protein for low‐sulfated KS.

## 2 Materials and Methods

### 2.1 Human Tissue Samples

Formalin‐fixed, paraffin‐embedded tissue blocks of MPM (*n* = 41) were retrieved from the pathology archive of University of Fukui Hospital and Hospital of Hyogo College of Medicine. Fresh human normal pleural tissue was obtained at autopsy from two cadavers.

### 2.2 Deglycosylation Treatments

To remove *N*‐glycans, tissue sections were incubated with 5,000 U/mL of peptide‐*N*‐glycosidase F (PNGase F) from *Flavobacterium meningosepticum* (New England BioLabs, Ipswich, MA) at 37°C for 90 min in a humidified chamber, as described [9, 21]. To degrade KS, sections were treated with 50 µg/mL of recombinant keratanase II from *Bacillus circulans* (Tokyo Chemical Industry, Tokyo, Japan). To degrade poly‐LacNAc and low‐sulfated KS, sections were treated with 50 µg/mL of endo‐β‐galactosidase from *Citrobacter freundii*, purified as described [22, 23]. As positive controls for digestion, porcine corneal tissue sections rich in *N*‐glycosidically linked KS glycosaminoglycans were similarly treated with the above three endoglycosidases and immunostained with anti‐KS antibody 5D4 (see below) [9].

### 2.3 Monoclonal Antibodies

The following monoclonal antibodies served as primary antibodies: SKM9‐2 (mouse IgG; Nichirei Biosciences, Tokyo, Japan) recognizing human HEG1 modified with sialic acid‐containing glycans [7]; 5D4 (mouse IgG; Seikagaku Corporation, Tokyo, Japan) recognizing highly sulfated KS [24]; and R‐10G (mouse IgG; Tokyo Chemical Industry) [14, 15] and 294‐1B1 (mouse IgM) [17], both of which recognize low‐sulfated KS.

### 2.4 Immunohistochemistry

Immunohistochemical staining was undertaken using the Histofine system (Nichirei Biosciences), as per the manufacturer's protocol. Immunoreactivity was considered positive if ≥ 10% of tumor cells were stained, as recommended by guidelines from the International Mesothelioma Interest Group [25]. Moreover, in the present study, cases in which less than 10% of tumor cells (but at least one cell) were stained were considered equivocal. On the other hand, cases in which none of the tumor cells were stained were judged negative.

### 2.5 Structural Analysis of Pleural KS/Sulfated LacNAc

Pleural KS/sulfated LacNAc was isolated and analyzed as described previously [26]. Post‐mortem human normal pleural tissue fragments (*n* = 4, weighing 100–320 mg) were homogenized in 2 mL of acetone, and the homogenate was then centrifuged and the pellet subjected to β‐elimination reaction and DNase/RNase treatment. Samples were then treated with actinase E (0.08 mg/mL) (Kaken Pharmaceutical, Tokyo, Japan) at 37°C overnight. KS/sulfated LacNAc was purified by diethylaminoethanol Sepharose column chromatography, and glycans were subjected to neuraminidase and fucosidase treatment. KS/sulfated LacNAc was then ethanol‐precipitated and digested overnight at 37°C with 0.5 mU keratanase II from *Bacillus* sp. Ks 36 (Seikagaku Corporation). Oligosaccharide composition of glycans was determined by reversed‐phase ion‐pair HPLC with post‐column fluorescent labeling, as described previously for chondroitin sulfate [27]. Authentic sulfated oligosaccharide markers were used to identify oligosaccharide components containing GlcNAc(6S) [28].

### 2.6 Construction of the HEG1·IgG Expression Vector

DNA fragments encoding the extracellular domain (amino acid residues 1–1,348) of human HEG1 were PCR‐amplified with the oligonucleotides 5′‐ACCCAAgCTggCTAgCCgCCgTCACCATggCCTC‐3′ and 5′‐CTCACCCTCgggATCCTgATAggggTTTCCACAgT‐3′ (*Nhe*I and *Bam*HI sites underlined) using Flexi ORF clone pF1KA1237 (Kazusa DNA Research Institute, Kisarazu, Japan), which harbors human HEG1 cDNA, as template. PCR products were inserted into *Nhe*I/*Bam*HI sites of pcDNA3.1/Hygro‐IgG [29] using In‐Fusion Snap Assembly Master Mix (Takara Bio, Kusatsu, Japan), resulting in pcDNA3.1/Hygro‐HEG1·IgG.

### 2.7 Low‐Sulfated KS‐Expressing Cell Lines and Western Blot Analysis of HEG1·IgG Glycoforms

Human embryonic kidney (HEK) 293 T/GC cells expressing low‐sulfated KS were established previously [17, 30]. Briefly, HEK 293T cells (cultured in Dulbecco's modified Eagle's medium supplemented with 10% fetal bovine serum) were transfected with pCDH lentiviral vectors carrying cDNAs encoding enzymes essential for low‐sulfated KS biosynthesis, i.e. β1,3‐*N*‐acetylglucosaminyltransferase 7 (β3GlcNAcT‐7) and corneal GlcNAc‐6‐*O*‐sulfotransferase (C‐GlcNAc6ST). Cells were transiently transfected with pcDNA3.1/Hygro‐HEG1·IgG and cultured in HyClone SFM4Transfx‐293 serum‐free medium (Thermo Fisher Scientific, Waltham, MA) supplemented with 4 mM l‐glutamine for 48 h. Protein A Sepharose Fast Flow (Sigma‐Aldrich, St. Louis, MO) was added to the conditioned medium, and incubated at 4°C for 60 min on a rotator. Beads were washed 5 times with phosphate‐buffered saline, treated with sample buffer, and then subjected to western blot analysis, as described [31, 32]. Briefly, after incubation at 100°C for 2 min, samples were subjected to sodium dodecyl sulfate‐polyacrylamide gel electrophoresis and transferred onto a polyvinylidene difluoride membrane. After blocking, the membrane was incubated with 294‐1B1 or R‐10G antibodies at 4°C overnight. After washing, membrane was incubated with horseradish peroxidase (HRP)‐conjugated anti‐mouse IgM or IgG (Jackson ImmunoResearch, West Grove, PA) for 60 min. To detect HEG1·IgG fusion proteins, the membrane was incubated directly with HRP‐conjugated anti‐human IgG (Jackson ImmunoResearch). Immunoreactive bands were visualized using SuperSignal West Dura Extended Duration Substrate (Thermo Fisher Scientific) and a luminescent image analyzer LAS‐4000 (GE Healthcare, Chicago, IL).

## 3 Results

### 3.1 Expression of Low‐Sulfated KS in Human Normal Pleural Mesothelium

**Figure 1:**
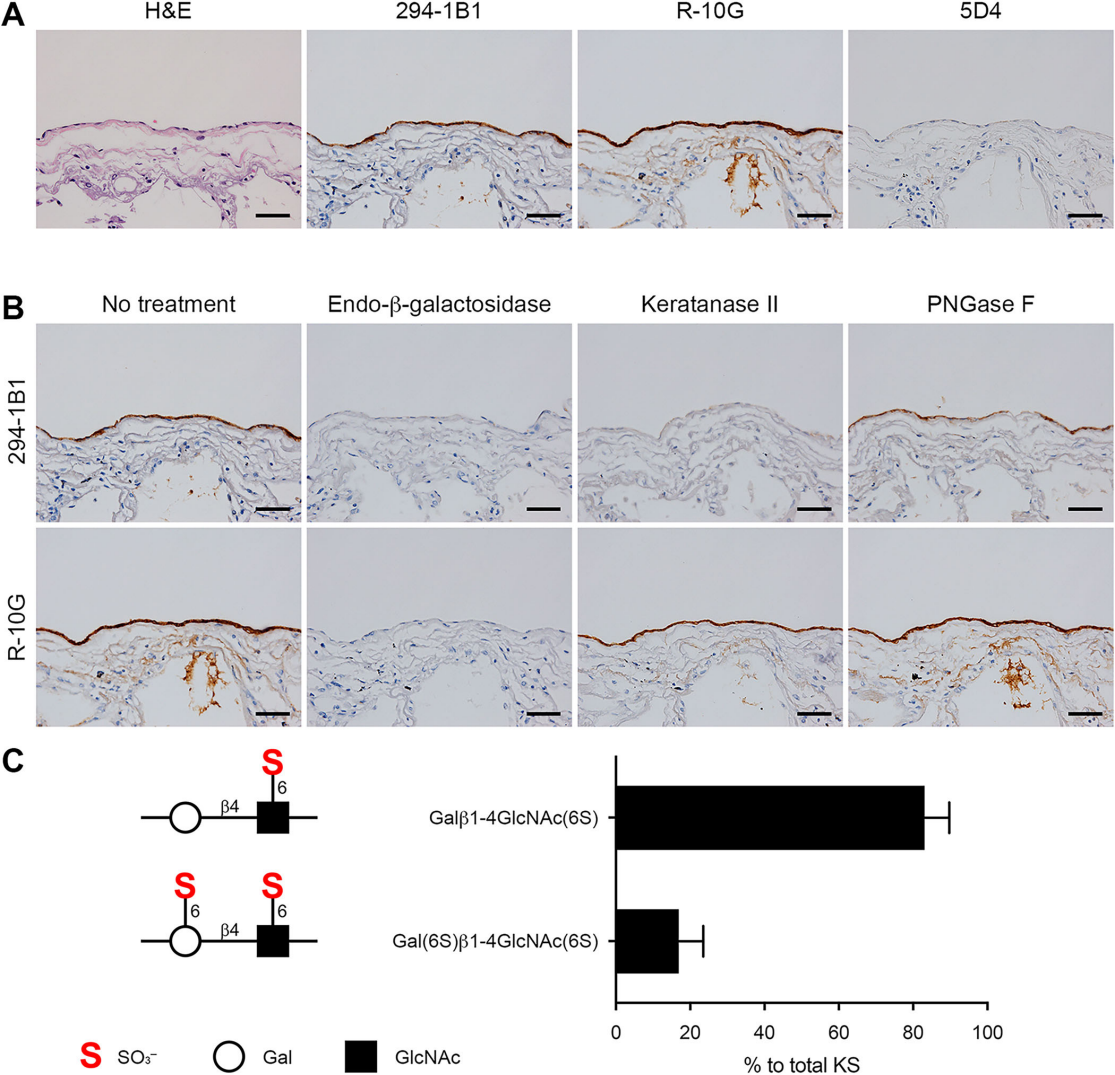
Expression of low‐sulfated KS in human pleural mesothelium. (A) Immunohistochemical profiles of KS expressed in human normal pleural mesothelium. Serial tissue sections were stained with hematoxylin and eosin (H&E) or immunostained for 294‐1B1, R‐10G or 5D4. (B) Susceptibility of low‐sulfated KS expressed in human normal pleural mesothelium to a series of endoglycosidases. Serial tissue sections were treated with or without indicated endoglycosidases and then immunostained for 294‐1B1 or R‐10G. Photomicrographs in the left column are the same as their counterparts shown in (A). In (A) and (B), signals were visualized with 3,3′‐diaminobenzidine (brown), and tissues were counterstained with hematoxylin. Bar = 50 µm. (C) Disaccharide compositions of KS/sulfated LacNAc purified from fresh human normal pleural tissue, as revealed by reversed‐phase ion‐pair HPLC analysis. The percentages of Galβ1‐4GlcNAc(6S) (upper bar) and Gal(6S)β1‐4GlcNAc(6S) (lower bar) to total KS/sulfated LacNAc are shown as means with standard deviation. Each disaccharide structure is shown on the left.

To examine staining patterns in normal tissue, we first performed immunohistochemical staining of human normal pleura with anti‐KS antibodies. As shown in Figure 1A, mesothelial cells lining the pleura were stained with 294‐1B1 and R‐10G, while 5D4 immunolabeling was negligible, indicating that normal mesothelial cells primarily express low‐sulfated KS. Consistent with this finding, immunolabeling with both 294‐1B1 and R‐10G was completely eliminated by endo‐β‐galactosidase digestion (Figure 1B, second column). Interestingly, while 294‐1B1 signals were completely eliminated by keratanase II digestion, R‐10G signals were keratanase II‐resistant (Figure 1B, third column), consistent with previous analysis performed in mice [16]. We next carried out reversed‐phase ion‐pair HPLC analysis of KS/sulfated LacNAc purified from human normal pleural tissue. As shown in Figure 1C, Galβ1‐4GlcNAc(6S) mono‐sulfated disaccharide and Gal(6S)β1‐4GlcNAc(6S) di‐sulfated disaccharide accounted for 83.1% and 16.9% of total KS/sulfated LacNAc, respectively, a result in agreement with immunohistochemical findings.

### 3.2 Expression of Low‐Sulfated KS in MPM

**Figure 2:**
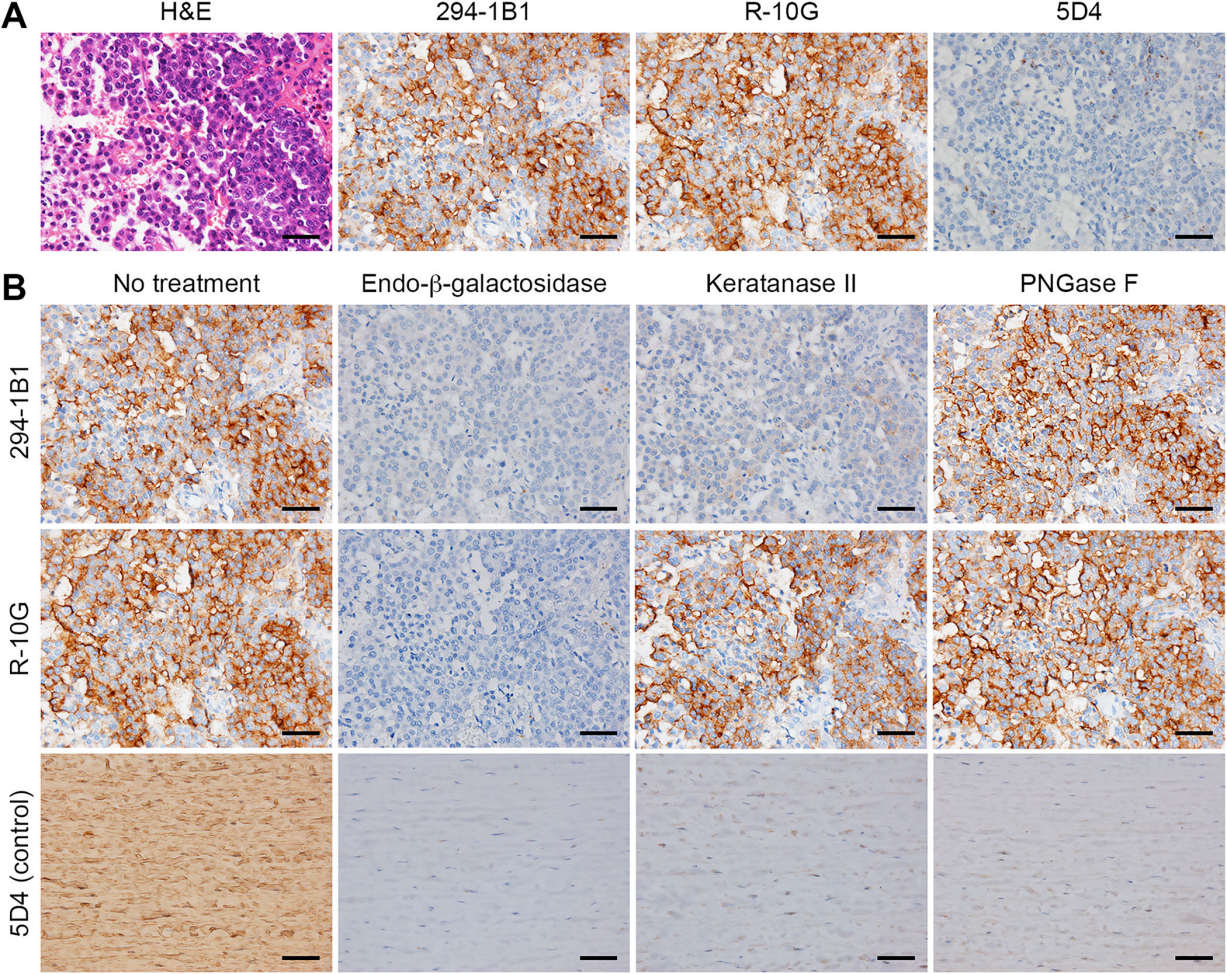
Expression of low‐sulfated KS in MPM. (A) Immunohistochemical profiles of KS expressed in a representative case of epithelioid type MPM. Serial tissue sections were stained with hematoxylin and eosin (H&E) or immunostained for 294‐1B1, R‐10G or 5D4. (B) Susceptibility of low‐sulfated KS expressed in MPM to a series of endoglycosidases. Serial tissue sections were treated with or without indicated endoglycosidases and then immunostained for 294‐1B1 or R‐10G. Photomicrographs in left top and middle panels are the same as their counterparts shown in (A). Control porcine corneal tissue sections were similarly treated and stained for 5D4. In (A) and (B), signals were visualized with 3,3’‐diaminobenzidine (brown), and tissues were counterstained with hematoxylin. Bar = 50 µm.

**Figure 3:**
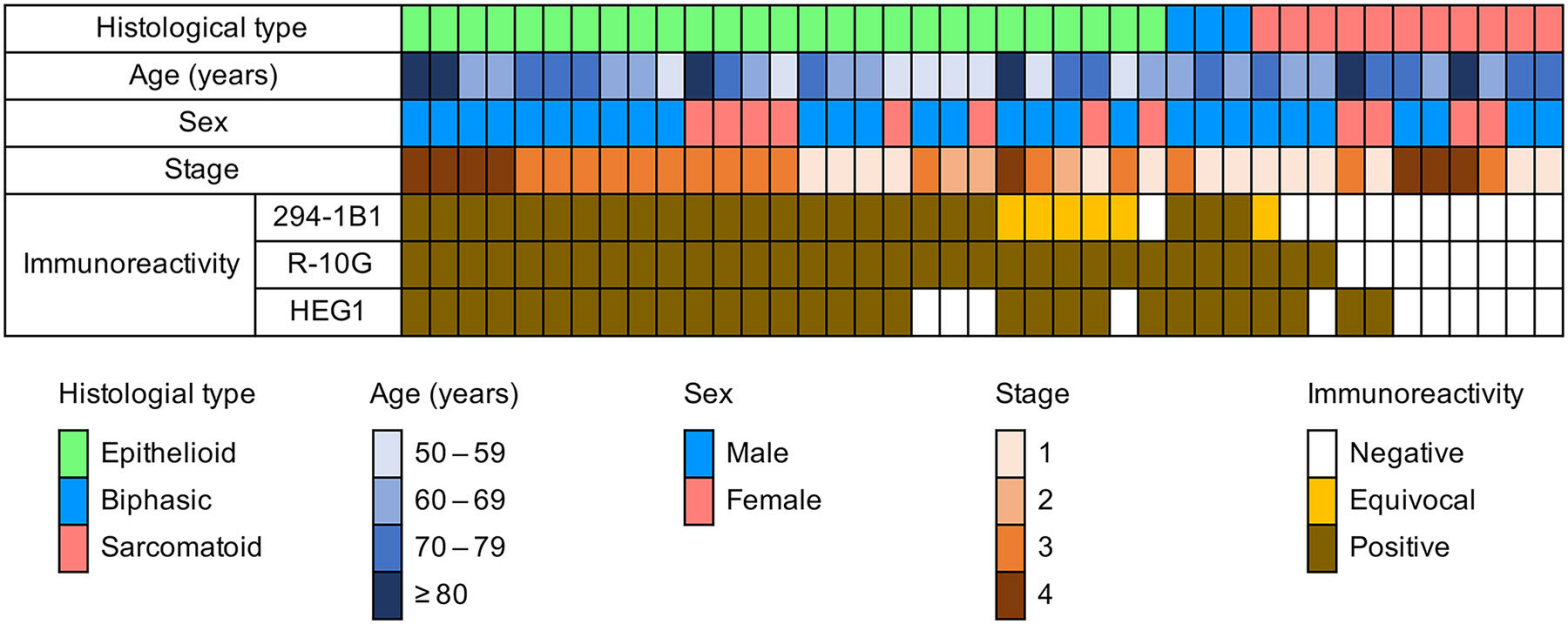
Clinicopathological characteristics and expression patterns of HEG1 and low‐sulfated KS recognized by 294‐1B1 and R‐10G in 41 cases of MPM examined in this study.

We next performed immunohistochemical staining of MPM with anti‐KS antibodies. As shown in Figures 2A and 3, 21 of 27 cases (77.8%) of epithelioid type MPM were 294‐1B1‐positive, and all cases (100%) were stained with R‐10G. The epithelioid component of biphasic type MPM was positive for both antibodies. By contrast, 5D4 immunolabeling in MPM was negligible. Similar to normal pleural mesothelium, 294‐1B1 and R‐10G signals were completely eliminated by endo‐β‐galactosidase digestion (Figure 2B, second column). On the other hand, while 294‐1B1 immunolabeling was abolished by keratanase II digestion, R‐10G immunolabeling was keratanase II‐resistant (Figure 2B, third column). As for sarcomatoid type MPM, none of 11 cases (0%) was 294‐1B1‐positive, and 3 of 11 cases (27.3%) were R‐10G‐positive (Figure 3). Taken together, these findings indicate that MPM, particularly the epithelioid type, expresses low‐sulfated KS, similar to normal pleural mesothelium.

### 3.3 Low‐Sulfated KS Expressed in MPM Is Carried on Core 2 *O*‐Glycans

We then digested both normal mesothelium and MPM tissue sections with PNGase F before immunostaining with anti‐KS antibodies. As shown in Figures 1B and 2B (both right column), 294‐1B1 and R‐10G signals were resistant to PNGase F digestion, while 5D4 immunolabeling in control porcine corneal tissue was eliminated by PNGase F digestion. Moreover, as we and others previously reported, immunolabeling of mesothelial cells with MECA‐79 monoclonal antibody, which recognizes GlcNAc‐6‐*O*‐sulfated LacNAc attached to extended core 1 *O*‐glycans [33, 34], was negligible in both normal mesothelium and MPM [9, 10]. These findings combined indicate that pleural low‐sulfated KS is most likely carried on core 2 *O*‐glycans.

### 3.4 HEG1 Serves as a Core Protein of Low‐Sulfated KS

**Figure 4:**
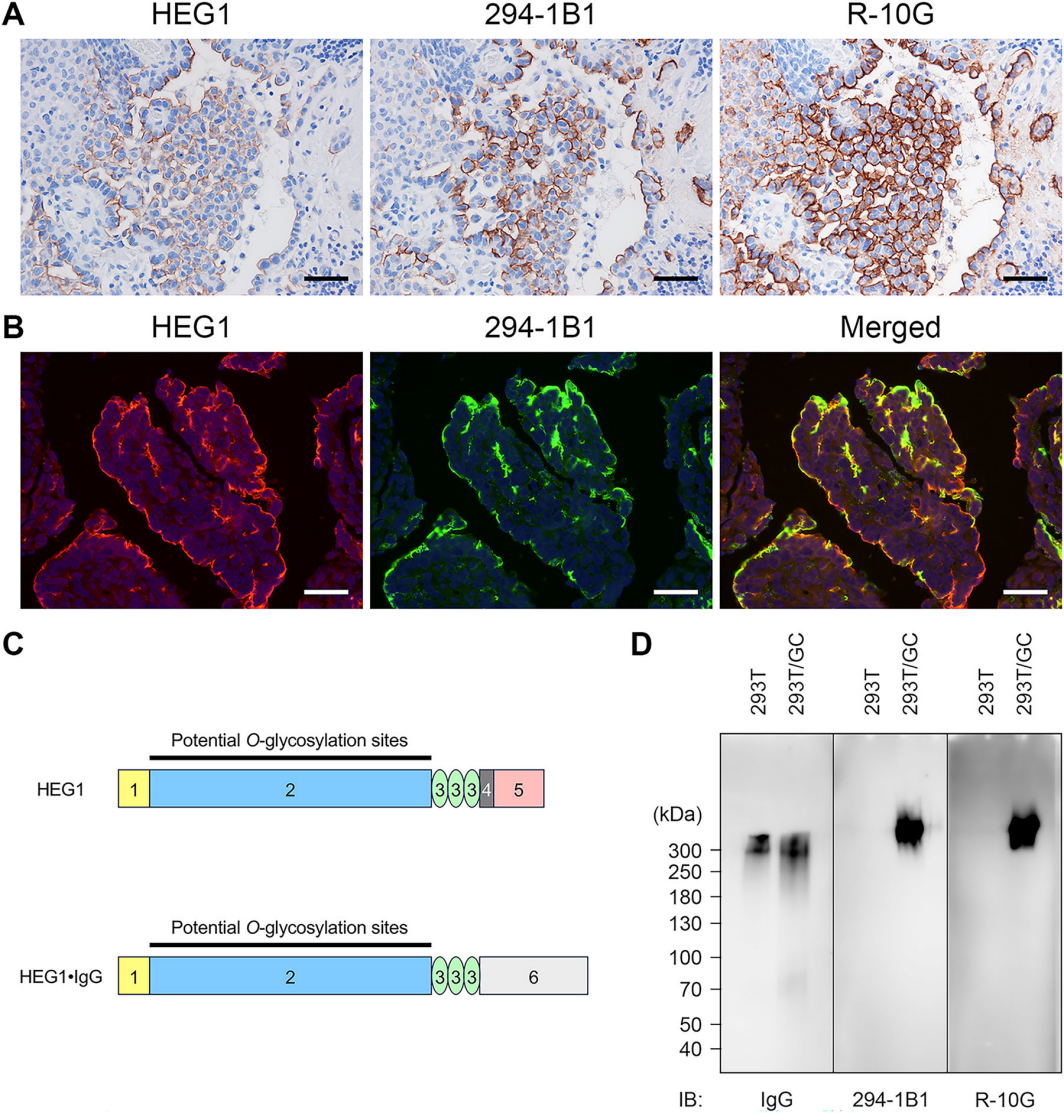
HEG1 functions as a core protein for low‐sulfated KS. (A) Expression patterns of HEG1 and low‐sulfated KS recognized by 294‐1B1 and R‐10G in a representative case of epithelioid type MPM. Serial tissue sections were immunostained for HEG1, 294‐1B1 or R‐10G. Signals were visualized with 3,3′‐diaminobenzidine (brown), and tissues were counterstained with hematoxylin. Bar = 50 µm. (B) Double immunofluorescence staining for HEG1 (red) and 294‐1B1 (green) on a representative case of epithelioid type MPM. Yellow signals in merged images indicate antigen co‐localization. Sections were counterstained with 4′,6‐diamidino‐2‐phenylindole (blue). Bar = 50 µm. (C) Schematic showing simplified structures of human HEG1 (upper) and HEG1·IgG fusion protein used in this study (lower). Numbers indicate the following: 1, proline‐rich domain; 2, serine/threonine‐rich region, which exhibits potential *O*‐glycosylation sites; 3, epidermal growth factor (EGF) domain; 4, transmembrane domain; 5, cytoplasmic domain; and 6, human IgG constant region. Based on ref. 7. (D) Western blot analysis of protein A‐purified human HEG1·IgG glycoforms secreted from HEK 293T/GC and parental 293T cells. Membrane was immunoblotted (IB'd) for IgG (left panel), 294‐1B1 (middle panel) or R‐10G (right panel). Numbers at left indicate molecular weight (kDa).

Since the staining patterns of HEG1 and low‐sulfated KS were similar to each other (Figures 3 and 4A), we hypothesized that HEG1 functions as a core protein for low‐sulfated KS. To test this hypothesis, we first performed double immunofluorescence staining of MPM tissue for HEG1 and 294‐1B1. As shown in Figure 4B, most HEG1 signals found on the apical membrane of tumor cells were colocalized with 294‐1B1 signals. We then performed western blot analysis of protein A‐purified HEG1·IgG fusion proteins (Figure 4C) secreted from low‐sulfated KS‐expressing HEK 293T/GC cells and control parental 293T cells. As shown in Figure 4D, immunoblotting for IgG (first and second lanes) revealed a band migrating at 300–350 kDa with a smear of 180–300 kDa in both 293T/GC and control 293T cells, indicating that HEG1·IgG fusion proteins were secreted from both cell lines. In contrast, immunoblots for 294‐1B1 (third and fourth lanes) and R‐10G (fifth and sixth lanes) showed a band migrating at 300–500 kDa only in low‐sulfated KS‐expressing 293T/GC cells (fourth and sixth lanes). This finding strongly suggests that HEG1 functions as a core protein for low‐sulfated KS.

## 4 Discussion

Here we demonstrate that low‐sulfated KS is expressed on human normal pleural mesothelial cells as well as MPM tumor cells, particularly of the epithelioid type, and that such low‐sulfated KS is likely displayed on core 2 *O*‐glycans. Moreover, our western blot analysis indicates that HEG1 functions as a core protein for low‐sulfated KS.

In this study, normal mesothelial cells were positive for 294‐1B1 and R‐10G but only minimally stained with 5D4. Moreover, immunolabeling with 294‐1B1 and R‐10G was completely abrogated by endo‐β‐galactosidase digestion. These findings combined indicate that KS expressed in pleural mesothelium is primarily of the low‐sulfated type and that highly sulfated KS, if present at all, is rare. To quantitatively analyze the proportion of low‐sulfated KS in total KS, we conducted reversed‐phase ion‐pair HPLC analysis. This analysis of disaccharide composition revealed that Galβ1‐4GlcNAc(6S) mono‐sulfated and Gal(6S)β1‐4GlcNAc(6S) di‐sulfated disaccharides accounted for 83.1% and 16.9%, respectively, of total KS/sulfated LacNAc present in human pleural tissue, consistent with our immunohistochemical results. Since keratanase II eliminates 294‐1B1 but not R‐10G signals in human pleural mesothelium, Galβ1‐4GlcNAc(6S) mono‐sulfated LacNAc disaccharide detected in this analysis is likely derived from 294‐1B1‐reactive low‐sulfated KS.

As noted, 294‐1B1 immunolabeling of mesothelial cells was completely abolished by keratanase II digestion, a finding consistent with the idea that 294‐1B1 recognizes low‐sulfated KS. By contrast, in comparable analysis, we observed R‐10G immunolabeling to be keratanase II‐resistant, indicating that R‐10G recognizes carbohydrate structures other than low‐sulfated KS. Previously, we observed a similar phenomenon in the context of ovarian cancer, in which signals for R‐10G, but not 294‐1B1, on human ovarian carcinoma OVCAR‐3 cells were not completely eliminated by keratanase II digestion [17]. These findings collectively indicate that R‐10G recognizes not only genuine low‐sulfated KS but also related structures resistant to keratanase II digestion. The R‐10G epitope has been shown to be GlcNAc‐6‐*O*‐sulfated di‐LacNAc tetrasaccharide, Galβ1‐4GlcNAc(6S)β1‐3 Galβ1‐4GlcNAc(6S) [15], which overlaps with the keratanase II‐sensitive glycosequence. Thus, theoretically, the R‐10G epitope should be eliminated by keratanase II digestion. A possible explanation for this phenomenon is that additional modifications, such as fucosylation and/or sialylation, of low‐sulfated KS do not inhibit R‐10G antibody binding to its epitope but do inhibit access of the keratanase II catalytic pocket to its substrate's glycosequence.

We previously demonstrated that sialyl GlcNAc‐6‐*O*‐sulfated LacNAc‐capped core 2 *O*‐glycans recognized by the S1 monoclonal antibody [11] were expressed in MPM and in normal pleural mesothelium [9]. However, it is not yet clear whether this structure is based on a poly‐LacNAc structure. Takeda‐Uchimura et al. previously demonstrated that mouse pleural mesothelium stained positively for the CL40 monoclonal antibody, which recognizes sialyl GlcNAc‐6‐*O*‐sulfated LacNAc [12], and that CL40 signals on the pleural mesothelium were resistant to endo‐β‐galactosidase digestion [10]. This finding suggests that CL40‐reactive glycans on the pleural mesothelium are sialyl GlcNAc‐6‐*O*‐sulfated mono‐LacNAc without poly‐LacNAc extension. On the other hand, in the present study, we observed that human pleural mesothelium stained positively with 294‐1B1 and R‐10G antibodies and that these signals were completely lost upon endo‐β‐galactosidase digestion, indicating the presence of at least two LacNAc repeats. These results suggest overall that sialyl GlcNAc‐6‐*O*‐sulfated poly‐LacNAc expressed on mesothelial cells, particularly in mice, contains a varying number of LacNAc repeats, with sialyl GlcNAc‐6‐*O*‐sulfated mono‐LacNAc likely the predominant one.

Western blot analysis of HEG1·IgG secreted from low‐sulfated KS‐expressing HEK 293T/GC cells indicated that the molecular weight of HEG1·IgG decorated with low‐sulfated KS (300–500 kDa) was greater than that of unglycosylated or underglycosylated forms (180–350 kDa). This finding is consistent with the fact that the molecular weight of the HEG1·IgG fusion protein used here is calculated to be 165 kDa in its unglycosylated state, and that this protein harbors serine/threonine‐rich potential *O*‐glycosylation sites (see Figure 4C). These serine/threonine‐rich regions are presumably *O*‐glycosylated with low‐sulfated KS, resulting in formation of higher molecular weight HEG1·IgG glycoforms.

Mesothelial cells are characterized by abundant brush‐like microvilli rich in sialomucin and/or hyaluronic acid, which function as a lubricant between the lungs and thorax and reduce friction resulting from continuous breathing movements [11, 35]. In addition to the above‐noted hydrating molecules, the negative charge of sulfate groups in low‐sulfated KS likely contributes to this function. Expression of low‐sulfated KS seen in normal mesothelial cells was preserved even after malignant transformation; however, it is not known whether expression of low‐sulfated KS contributes to malignancy.

In conclusion, here we demonstrate that HEG1 protein expressed in MPM is likely decorated with low‐sulfated KS. This HEG1 glycoform may be a potential target for new therapies such as NIR‐PIT. Development of such minimally invasive MPM treatment will require generation of monoclonal antibodies selectively recognizing HEG1 decorated with low‐sulfated KS and their validation in animal models of MPM.

## Author Contributions

All authors contributed to this article as follows: Koki Nakashima designed and performed the research, analyzed the data, and wrote the manuscript; Hitomi Hoshino performed the research; Zui Zhang performed the research; Tomoya O. Akama designed and performed the research; Nobuyuki Kondo performed the research, Seiki Hasegawa contributed essential samples; Yoshitaka Sekido analyzed the data and revised the manuscript for important intellectual content; Mana Fukushima designed the research; Tamotsu Ishizuka organized the research team; and Motohiro Kobayashi conceived of and designed the research, analyzed the data, and wrote the manuscript. All authors have read and approved the final manuscript.

## Ethics Statement

This study was approved by the Research Ethics Committee of University of Fukui (reference number 20200045, approved on July 24, 2020) and by a comparable committee at Hyogo College of Medicine (reference number 3705, approved on January 22, 2021).

## Conflicts of Interest

The authors declare no conflicts of interest.

## Data Availability

The data that support the findings of this study are available from the corresponding author upon reasonable request.
